# Low Serum Paraoxonase-1 Activity Associates with Incident Cardiovascular Disease Risk in Subjects with Concurrently High Levels of High-Density Lipoprotein Cholesterol and C-Reactive Protein

**DOI:** 10.3390/jcm8091357

**Published:** 2019-09-01

**Authors:** James P. Corsetti, Charles E. Sparks, Richard W. James, Stephan J. L. Bakker, Robin P. F. Dullaart

**Affiliations:** 1 Department of Pathology and Laboratory Medicine, University of Rochester School of Medicine and Dentistry, Rochester, NY 1211, USA; 2 Department of Medical Specialties-Endocrinology, Diabetology, Hypertension and Nutrition, University of Geneva, 1201 Geneva, Switzerland; 3 Department of Nephrology, University of Groningen and University Medical Center Groningen, 9700 RB Groningen, The Netherlands; 4 Department of Endocrinology, University of Groningen and University Medical Center Groningen, 9700 RB Groningen, The Netherlands

**Keywords:** cardiovascular disease, C-reactive protein, HDL, paraoxonase-1

## Abstract

Paroxonase-1 (PON1) is a key enzyme that inhibits low-density lipoprotein oxidation and consequently atherogenesis. Here, we assessed whether low serum PON1 activity associates with incident cardiovascular disease (CVD) in subjects with high levels of high-density cholesterol (HDL-C) and C-reactive protein (CRP), a marker of low-grade systemic inflammation. Cox proportional-hazards modeling of incident CVD risk (11 years mean follow-up) adjusted for relevant clinical and biomarker covariates was performed on a population-based study (N = 7766) stratified into three groups: low CRP—(LR; event rate 4.9%); low HDL-C/high CRP—(HR1; event rate 14.4%); and high HDL-C/high CRP—(HR2; event rate 7.6%). Modeling results for PON1 activity in HR2 were significant and robust (hazard ratio/SD unit—0.68, 95% CI 0.55–0.83, *p* = 0.0003), but not so for LR and HR1. Analyses in HR2 of the interaction of PON1 with HDL-C, apoA-I, apoA-II, and apoE levels were significant only for PON1 with apoE (hazard ratio—1.77, 95% CI 1.29–2.41, *p* = 0.0003). Subsequent subgroup analysis revealed inverse risk dependence for apoE at low PON1 levels. In conclusion, in a population-based study of subjects with concurrently high HDL-C and CRP levels, low serum PON1 activity associates with incident CVD risk with risk accentuated at low apoE levels.

## 1. Introduction

Paraoxonases make up a family of three enzymes (PON1, PON2, and PON3) with predominantly lactonase activity, each of which has been shown to manifest anti-atherogenic properties. This presumably relates to hydrolytic activity against lactone-like structures elaborated by oxidized polyunsaturated fatty acids on lipoproteins [1,2]. PON2 is an intracellular enzyme. After synthesis in the liver, PON1 and PON3 enter the circulation and become predominantly associated with high-density lipoprotein (HDL) particles. Anti-atherogenic action of PON1 has been demonstrated to derive in large part from the interaction of HDL-associated PON1 with low-density lipoprotein (LDL) particles. Such interaction leads to inhibition of oxidative modification of LDL through PON1-mediated hydrolysis of oxidized fatty acid species derived from oxidative stress-induced generation of hydroperoxides from phospholipids, cholesteryl esters, and triglycerides [2,3,4,5,6]. Likewise for HDL, studies have also demonstrated similar anti-oxidative activity by PON1 on lipid peroxidation products generated on HDL in oxidative stress environments [7,8,9,10]. The putative anti-atherogenic properties of PON1 stem from meta-analyses of human population studies that consistently show decreased PON1 activity to be associated with cardiovascular disease (CVD) risk [11,12,13]. It should also be noted that in addition to CVD, low PON1 activity has been reported in other disease states having a significant inflammatory component including diabetes mellitus, obesity, metabolic syndrome, cancer, and various rheumatic, renal, hepatic, and neurologic diseases [3,14].

Accumulating evidence demonstrates that the established anti-atherogenic actions of HDL become dysfunctional and potentially pro-atherogenic in chronic inflammatory and oxidative stress environments as a result of ensuing alterations in the HDL proteome and lipidome [15,16,17]. To elucidate the role of dysfunctional HDL in CVD risk, investigations of HDL-associated risk have been undertaken in populations with various inflammatory disorders. Notably, results from such studies demonstrate that high levels of HDL cholesterol (HDL-C) associate with increased CVD risk rather than with protective effects [18]. In our case, we have studied incident as well as recurrent coronary events in subjects with high HDL-C and concurrently high levels of C-reactive protein (CRP) as a marker of low-grade chronic inflammation. Compared to the controls, the results demonstrated significantly higher CVD risk in these patient groups [19,20]. Furthermore, within these populations, additional studies demonstrated risk associations for levels of apolipoprotein E (apoE) [21] and apolipoprotein A-II (apoA-II) [22]; and, in studies of single nucleotide polymorphisms, risk associations were found for genes associated with various aspects of HDL function including reverse cholesterol transport [19,23], thrombogenesis [24], oxidative stress [25], and fibrinolysis [26].

In light of the key role of PON1 in inhibiting oxidative changes in the HDL lipidome that acts to preserve anti-atherogenic functionality of HDL as described above, we hypothesized that low PON1 levels in subjects with high levels of HDL-C in the setting of chronic low-level inflammation would be at increased risk for CVD. To test this hypothesis, we studied risk of incident CVD in subjects having concurrently high HDL-C and CRP levels as an indicator of chronic low grade inflammation in comparison to two other subject groups, one with low HDL-C and high CRP levels and the other with “normal” HDL-C and low CRP levels. The present study was carried out in the frame of the prospective Prevention of Renal and Vascular End-stage Disease (PREVEND) cohort study.

## 2. Experimental Section

### 2.1. Study Population

PREVEND, a large general population-based prospective cohort study begun in 1997 that aimed to explore the role of albuminuria in cardiovascular and renal disease, served as the basis of the current study population [27]. Briefly for PREVEND recruitment, all inhabitants of the city of Groningen, the Netherlands, aged 28–75 years (N = 85,421), were sent a questionnaire requesting an early morning urine specimen. The response rate was 48% (N = 40,856). Of these, 9966 subjects had urine albumin levels ≥10 mg/L while 30,890 subjects had urine albumin <10 mg/L. Exclusions included pregnancy, type 1 diabetic individuals, and type 2 diabetic individuals using insulin. This resulted in 7768 subjects having urine albumin ≥10 mg/L. This group was combined with 3395 randomly chosen subjects with urine albumin <10 mg/L. Of this resultant group, 8592 individuals completed an additional screening program, thus establishing the PREVEND study cohort. For the present work, the only further exclusion from the PREVEND study cohort was for subjects with serum CRP levels ≥10 mg/L. This formed the study population of the current work (N = 7766). The PREVEND study was approved by the Medical Ethics Committee of the University of Groningen, the Netherlands. Informed written consent was obtained from all participants. The study adhered to the ethical principles set by the Declaration of Helsinki.

### 2.2. Clinical Parameters and Biomarkers

Questionnaires were used to ascertain cardiac history (hospitalization for myocardial infarction, revascularization procedures, or obstructive coronary artery disease). Diabetes was established either as fasting plasma glucose ≥7.0 mmol/L, self-report of physician diagnosis, or use of anti-diabetic medications. Medication use was retrieved via Groningen pharmacy dispensing data [28]. Smoking status was either current smoker or not; ethanol use was either one or less drinks per day or more than one drink per day (with one drink assumed to contain 10 g of alcohol). Body mass index (BMI) was calculated as weight divided by height squared (kg/m^2^). Blood pressure was measured for 10 min at 1-min intervals using automatic instrumentation (Dinamap XL Model 9300; Johnson-Johnson Medical, Tampa, FL, USA) in the supine position; means of the last two recordings were the reported values [29].

The mean of two 24 h collections over two consecutive days at study entry was determined and used as the reported UAE value. Nephelometry (BNII, Dade Behring, Marburg, Germany) was used to measure urinary albumin concentration. Blood biomarker levels at study initiation were performed on overnight-fasted plasma and sera. Blood levels of creatinine, HDL-C, apoA-I, apoA-II, apoE, total cholesterol, triglycerides, apoB, glucose, and CRP were determined by standard techniques [20,21,30]. Estimated glomerular filtration rate (eGFR) was determined using the Chronic Kidney Disease Epidemiology Collaboration (CDK-EPI) equation [31]. NonHDL-C was calculated as the difference between total cholesterol and HDL-C. The Friedewald equation was used to estimate LDL-C levels. Serum PON1activity was measured in terms of arylesterase activity (rate of phenyl acetate hydrolysis to phenol) as described previously with inter-assay and intra-assay coefficients of variation of 8% and 6%, respectively [32].

### 2.3. Outcomes

CVD-related mortality and hospitalizations including acute MI (myocardial infarction), acute and subacute ischaemic heart disease, coronary artery bypass grafting, and percutaneous transluminal coronary angioplasty constituted incident cardiovascular events. Outcome events were followed over time (11 years of follow-up). Survival time was taken as date of initial urine collection (1997–1998) to date of first CVD event or to December 31, 2011. For subjects lost to follow-up (396 of the overall cohort of 8592 participants), censoring date was date of removal from the municipal registry. Record linkage with the Dutch Central Bureau of Statistics was used to acquire data on mortality and causes of death. PRISMANT, the Dutch national registry of hospital discharge diagnoses was used to obtain CVD morbidity data.

### 2.4. Data Analysis

Graphical and statistical analyses were performed using Statistica 13.3 (StatSoft, Inc., Tulsa, OK, USA). Graphical analyses portraying CVD risk over a bivariate parameter risk domain was performed using outcome event mapping as described previously [20]. Results are reported as means ± SD for normally distributed variables and as medians (interquartile range) for non-normally distributed variables. Differences among groups were assessed by ANOVA for continuous variables using Bonferroni correction for multiple comparisons and chi-square testing for categorical variables. Non-normal variables were log-transformed for analyses. Multivariable Cox proportional hazards modeling was used to follow cardiovascular outcomes over time. Continuous independent variables were standardized by transformation to distributions with means of zero and standard deviations of one for Cox analyses. Hazard ratios (HR) are per SD unit. Inclusion of multiplicative interaction terms into base models was used to assess interactions between risk variables. Two-sided *p*-values < 0.05 were considered statistically significant. The proportional hazards assumption was verified by correlation analysis of survival time with scaled Schoenfeld residuals.

## 3. Results

### 3.1. Study Population

**Figure 1:**
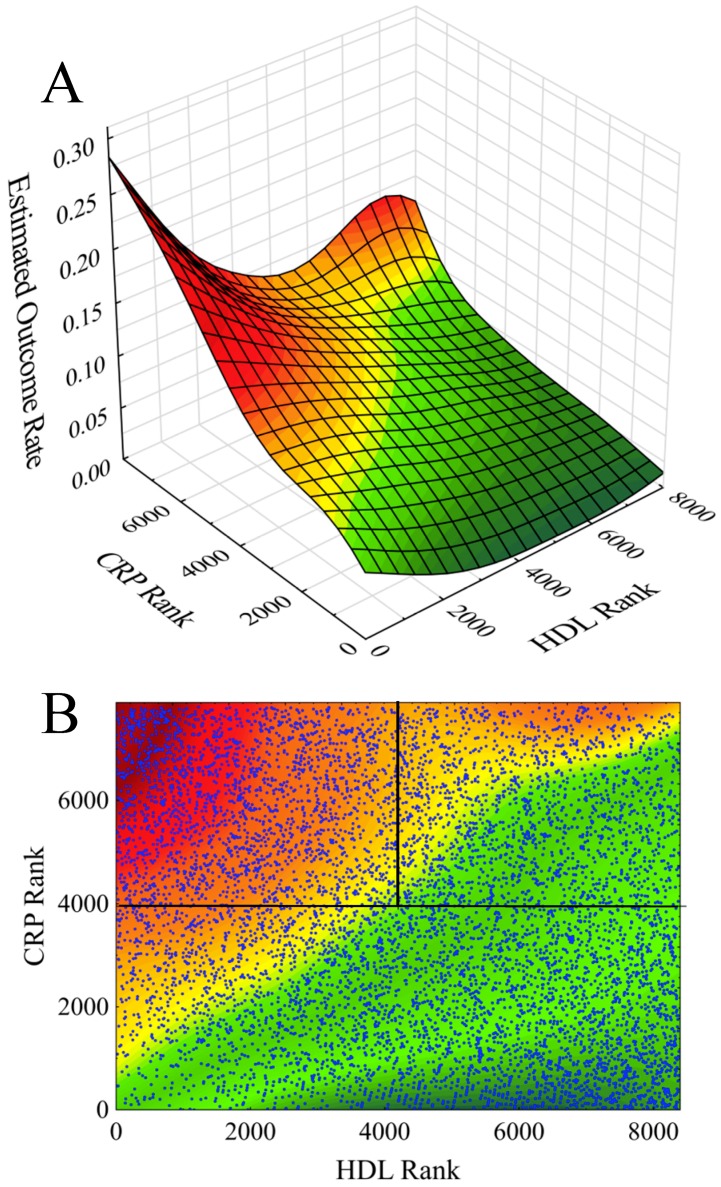
Cardiovascular event rates as a function of high-density lipoprotein (HDL) rank and C-reactive protein (CRP) rank given: (**A**) as a surface plot and (**B**) as a contour plot. The contour plot demarcates a low risk population (LR) characterized by lower CRP, a high-risk population (HR1) characterized by low HDL and high CRP, and another high-risk population (HR2) characterized by high HDL and high CRP.

During follow-up (11 years), a total of 643 events were recorded. The surface plot of Figure 1A demonstrates the estimated CVD outcome event rate as a function of HDL-C rank and CRP rank for the resultant total study population. The figure shows two peaks indicative of higher-risk populations: one at low HDL-C and high CRP rank and one at high HDL-C and high CRP rank. The corresponding contour plot of Figure 1B demonstrates the delineation of three populations based upon dichotomizations about median values of HDL-C and CRP ranks. The three resulting populations were designated as follows: low CRP—low risk (LR; N = 3889; 192 events; 4.9% event rate); low HDL-C/high CRP—high risk 1 (HR1; N = 2294; 331 events; event rate 14.4%); and high HDL-C/high CRP—high-risk 2 (HR2; N = 1583; 120 events; event rate 7.6%). Clinical and blood biomarker characterizations for the total, LR, HR1, and HR2 populations are given in Table 1. Results of chi square testing, unadjusted ANOVA, and ANOVA adjusted for gender and age revealed significant differences among the LR, HR1, and HR2 groups for all variables except ethanol use. Subsequent post-hoc testing revealed pair-wise significant differences between LR, HR1, and HR2 for all continuous variables except for: nonHDL-C (LR versus HR2, *p* = 0.078), triglycerides (LR versus HR2, *p* = 0.90), and UAE (LR versus HR2, *p* = 0.55).

### 3.2. Correlation of PON1 Activity with Blood Biomarker Levels

To provide further characterization of the study populations focusing on PON1 activity, Table 2 gives Spearman correlation coefficients for PON1 activity levels with blood biomarkers for the total, LR, HR1, and HR2 populations. The table shows that although many of the correlations are statistically significant, magnitudes of the coefficients are generally small except for HDL-C, apoA-I, and apoA-II (highest at 0.28). Thus, PON1 activity did not appear to be highly correlated with any of the blood biomarkers in any of the populations which may be indicative potentially of a relatively independent role of PON1 activity in CVD risk.

### 3.3. PON1 Activity as a Marker of CVD Risk in LR, HR1, and HR2 Populations

To assess PON1 activity as a marker of CVD risk, Table 3 gives results of Cox multivariable proportional hazards modeling in the three populations. For the LR population, PON1 activity was significant (protective) in an unadjusted model, but lost significance upon adjustment for gender and age. For HR1, PON1 activity, even in an unadjusted model, did not achieve significance. For HR2, PON1 activity was highly significant (protective) in an unadjusted model (*p* = 10^−6^); continued to be highly significant in a collection of models adjusted for gender, age, and a set of covariates added one-at-time; and finally remained significant (*p* = 0.0003) in a model adjusted simultaneously for all covariates of the dataset (gender, age, UAE, past CVD, DM, apoB, use of statins, anti-hypertensives, smoking, ethanol use, and eGFR). Thus, PON1 activity appears to have a robust inverse relationship with CVD risk in the HR2 population.

### 3.4. HDL Particle Constituents in Addition to PON1 as Markers of CVD Risk in HR2

To assess HDL-associated markers of risk in HR2 beyond PON1, we determined proportional hazards modeling in HR2 as a function of PON1 activity along with additional single entry of HDL-associated parameters (apoA-I, apoA-II, apoA-I/HDL-C, apoA-II/HDL-C, and apoE levels) (Table 4). Models also included evaluation of the potential interaction of PON1 activity with each of the HDL-associated parameters. Again, all models were adjusted for gender, age, UAE, past CVD, DM, apoB, use of statins, anti-hypertensives, smoking, ethanol use, eGFR, SBP, and DBP. The results from Table 4 indicate that, in each case for models without interaction, an independent significant protective association continued for PON1. For HDL-associated parameters, an independent protective association was demonstrated for apoA-I/HDL-C. In models assessing interactions, only apoE levels were found to exhibit a statistically significant interaction with PON1 activity (HR–1.77, 95% CI 1.29–2.41, *p* = 0.0003).

**Figure 2:**
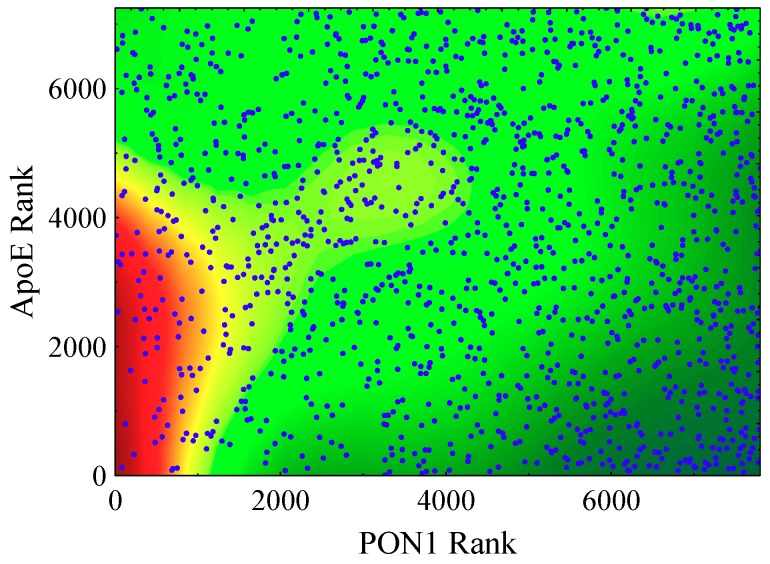
Contour plot of the estimated cardiac event rate as a function of PON1 rank and apoE rank in the high HDL/high CRP population (HR2).

To explore the nature of the PON1/apoE interaction in HR2, subgroup analysis using the proportional hazards model adjusted as before was performed on subjects of HR2 but this time with dichotomization about median PON1 levels giving low- and high-PON1 groups. Results indicated a trend in continued risk association with low PON1 levels both in the low PON1 group (HR—0.74, 95% CI 0.53−1.05, *p* = 0.089) and the high PON1 group (HR—0.60, 95% CI 0.26−1.40, *p* = 0.24). Regarding apoE and risk, while results for the high PON1 group demonstrated the generally expected trend in risk associated with high apoE levels (HR—1.41, 95% CI 0.91−2.18, *p* = 0.13), results for the low PON1 group revealed significant risk in association with low apoE levels (HR—0.69, 95% CI 0.50−0.96, *p* = 0.029). To illustrate the interaction, a contour plot of estimated CVD risk as a function of PON1 rank and apoE rank was generated (Figure 2). Consistent with the modeling results, the plot shows a high-risk population at concurrently low levels of both PON1 and apoE; while by contrast, at high PON1 levels, there is little indication of risk in association with low apoE levels. Hence in HR2, it would appear that the PON1/apoE interaction manifests as intensification of the usual CVD risk association for low PON1 activity but particularly in the setting of low apoE levels.

## 4. Discussion

Results of the current study of incident CVD show a highly significant association of low serum PON1 activity with risk in subjects with high levels of HDL-C in the setting of chronic low-grade inflammation, as indicated by high CRP levels. Furthermore, interaction analysis demonstrated such risk to be accentuated in those subjects with low levels of serum apoE; no corresponding effect was observed for apoA-I, apoA-II, or HDL-C levels within the high HDL-C/high CRP group. These results from multivariable proportional hazards modeling were robust with regard to adjustment for relevant clinical and blood biomarkers including gender, age, past CVD event, diabetes, apoB, statin use, anti-hypertensive use, smoking, ethanol use, and eGFR. It should be noted that corresponding analyses performed both on subjects with low HDL-C and high CRP and on subjects with low CRP did not demonstrate in either case statistically significant associations for PON1 activity with CVD. Our results suggest that the putative anti-oxidative effects of serum PON1 are most evidently exhibited in subjects with high PON1 and high HDL-C levels in the setting of chronic inflammation that is signaled by higher CRP levels.

Results of a recent meta-analysis addressing the role of PON1 activity on incident CVD risk demonstrated an inverse relationship between PON1 activity and risk in general population settings [13]. It was also noted in this study that the inverse relationship became less robust when adjustments for established risk factors including HDL-C were included—a finding that could potentially be relevant to our results regarding PON1 activity in the high HDL-C/high CRP group. However, this does not appear to be the case as results of risk models including PON1 activity and HDL-C levels were not significant for HDL-C (*p* = 0.82); nor was interaction between the two significant (*p* = 0.25). It should also be noted that corresponding analyses of PON1 activity with apoA-I and apoA-II were also non-significant. Accordingly, although study results show a significant inverse relationship for PON1 activity with risk, this is not the case for other HDL parameters including levels of HDL-C, apoA-I, and apoA-II. These findings underscore the key role of low PON1 activity in establishment of CVD risk in this subject group, which likely derives from inhibition of oxidative modification of the HDL lipidome, thus helping to maintain HDL anti-atherogenic functionality. Consistent with our results regarding low PON1 activity in the high HDL-C/high CRP subgroup, as noted previously, many other disease states associated with low-grade chronic inflammation also manifest low PON1 activity [3,14]. In the case of CVD risk, in the current study we have identified a group of subjects where PON1 action is particularly relevant.

Further results from our study did not show association of PON1 activity with risk both in the low risk subject group (low CRP levels) and in a subject group with low HDL-C/high CRP levels. In the first case, we speculate that the lower CRP levels provide an environment not conducive to oxidative modification of HDL, thus preserving HDL functionality and consequently limiting the relevance of PON1 activity in this group. In the second case, we speculate that higher PON1 was not protective in HR1 stemming in one way or another from the inadequacy of PON1 to significantly inhibit oxidation of the presumably heavier load of small dense LDL particles very likely present in this population enriched with Metabolic Syndrome subjects (Table 1; HR1—51.3%, HR2—9.7%, *p* < 0.001). Inadequacy of PON1 in this setting is likely attributable to multiple factors. First, the load of LDL-C in HR1 was significantly higher than in HR2 (Table 1; 4.04 mM U/L versus 3.58 mM U/L, *p* < 0.001); and thus, the concentration of potentially atherogenic dense LDL particles would be higher in HR1 given their well-established smaller size. Second, from the very defining characteristics of HR1, the level of HDL-C was less than in HR2 (Table 1; 1.01 mM versus 1.62 mM, *p* < 0.001). Thus, the amount of PON1, known to be predominantly carried by HDL, should be and was observed to be smaller in HR1 (Table 1; 50.8 U/L versus 56.1 U/L, *p* < 0.001). Third, the carriage ability of HDL particles for PON1 might be less in HR1 versus HR2 subjects. Thus, using the apoA-I/HDL-C ratio as a crude measure of the number of apoA-I molecules per HDL particle, the observed higher apoA-I/HDL-C ratio in HR1 versus HR2 (Table 1, 43.2 µM/mM versus 34.1 µM/mM, *p* < 0.001) would tend to limit carriage of additional molecules like PON1 on HDL particles (the situation was similar for the apoA-II/HDL-C ratio).

Additional results in the high HDL-C/high CRP group of our study using interaction analysis showed accentuation of CVD risk at concurrently low levels of PON1 and apoE. This is consistent with in vitro studies of PON1 associations with reconstituted HDL particles carrying apoE that show such binding to stabilize and stimulate PON1 lactonase activity as well as to stimulate PON1 anti-atherogenic potential in a manner similar to apoA-I but with lower capacity [33]. Presumably then, lower apoE levels would work to decrease PON1 anti-atherogenic action. Furthermore, results of a clinical study of metabolic syndrome (MetS) showed that although subjects without MetS demonstrated a positive correlation of PON1 activity with serum apoE levels, suggestive of enhancement of PON1 anti-atherogenic action with increased apoE levels; the relationship was abrogated in subjects with MetS, a condition associated with inflammation and oxidative stress [34].

There were strengths and limitations in our study. Strengths included the availability of a large well-characterized population-based study cohort followed prospectively for CVD outcomes for an average of 11 years and availability of extensive corresponding clinical and laboratory parameters including serum apoA-I, apoA-II, and apoE levels. The study was limited in a number of ways. In terms of the study population, there were exclusions for pregnancy, and type 1 diabetes mellitus and for subjects with serum CRP ≥10 mg/L (to minimize confounding effects by acute inflammation, rheumatic or other disease conditions manifesting high levels of inflammation); while it should also be noted that the study cohort was almost exclusively comprised of subjects of white European origin thus potentially limiting relevance of study findings to other populations. Additionally, the study population was enriched with albuminuric subjects; however, risk modeling results included adjustments for UAE and eGFR to account for this. In terms of clinical and laboratory parameters, values were determined only at study initiation, thus limiting the effects of parameter value variation over time. Regarding PON1 action, arylesterase activity as a measure was chosen because of good assay precision and good correlation with PON1 mass assays [35]. Lastly, there was no experimental evidence to verify that the HDL lipidome was actually preserved from oxidative dysfunctional transformation by high PON1 activity as speculated in our study. Poor performance of the technically difficult characterization of lipoprotein lipidomics might determine the effect of *PON1* on the risk of functional genetic polymorphisms.

In summary, the results of our current work show that low serum PON1 activity is associated with increased CVD risk in a group of subjects from a population-based study having concurrently high levels of HDL-C and CRP indicative of a chronic low-grade inflammatory environment. We believe that high serum PON1 activity in this group serves to facilitate inhibition of oxidative modification of the HDL lipidome, thus tending to preserve anti-atherogenic functionality of HDL particles including the curtailing of LDL oxidation. As such, these findings serve to underscore the presumptive efficacy of PON1 activity in the inhibition of lipoprotein oxidative modification.

## 5. Conclusion

Individuals having high HDL-C levels in the setting of chronic systemic inflammation have increased risk for incident cardiovascular disease. Furthermore, in such individuals, low serum PON1 levels associate with risk with risk accentuated at low apoE levels.” 

## Figures and Tables

**Table 1:** Clinical and biomarker parameters for the total study population and low risk (LR), high-risk 1 (HR1), and high-risk 2 (HR2) subpopulations. Biomarker levels are given as means ± standard deviations for normally distributed parameters and medians with interquartile ranges for non-normally distributed parameters.

Parameter	Total Population	Low Risk (86.1% of Subjects)		High-Risk 1		High-Risk 2		*p* ^a^	*p* ^b^
Subjects N (%)	7766	3889 (50.1)		2294 (29.5)		1583 (20.4)			
Outcome events N (%)	643 (8.3)	192 (4.9)		331 (14.4)		120 (7.6)		<0.001	
Age (years)	49.1 ± 12.6	46.2 ± 11.8		52.8 ± 12.5		50.9 ± 13.2		<0.001	
Females (%)	50.1	49.0		36.5		72.5		<0.001	
Cardiac history (%)	4.4	2.5		8.1		3.9		<0.001	
Diabetes (%)	3.2	1.4		7.0		2.1		<0.001	
Metabolic Syndrome	24.4	13.4		51.3		9.7		<0.001	
Statins (%)	4.1	3.1		6.3		3.3		<0.001	
Anti-hypertensives (%)	13.6	8.4		20.8		15.7		<0.001	
Current Smoker (%)	33.3	27.7		42.7		33.6		<0.001	
Ethanol Use (>1 drink/day %)	25.3	25.9		24.1		25.7		0.28	
Pulse Rate (per minute)	69.0	68.7		70.4		70.3		<0.001	<0.001
Systolic BP (mmHg)	129 ± 20	124 ± 18		135 ± 21		131 ± 22		<0.001	<0.001
Diastolic BP (mmHg)	74 ± 10	72 ± 9		77 ± 10		74 ± 10		<0.001	<0.001
BMI (kg/m^2^)	26.1 ± 4.2	24.6 ± 3.4		28.0 ± 4.3		26.2 ± 4.2		<0.001	<0.001
HDL-C (mM)	1.32 ± 0.40	1.40 ± 0.41		1.01 ± 0.18		1.62 ± 0.29		<0.001	<0.001
CRP (mg/L)	1.28 (0.56–2.98)	0.54 (0.30–0.82)		2.76 (1.79–4.62)		2.52 (1.71–4.16)		<0.001	<0.001
PON1 (U/L)	53.2 (43.2–65.1)	53.7 (43.7–65.6)		50.8 (40.9–62.0)		56.1 (46.6–68.3)		<0.001	<0.001
ApoA-I (μM)	47.8 ± 9.9	48.0 ± 10.0		42.8 ± 6.8		54.7 ± 9.3		<0.001	<0.001
ApoA-II (μM)	19.6 ± 3.8	19.5 ± 3.8		18.7 ± 3.2		21.4 ± 4.0		<0.001	<0.001
ApoA-I/HDL-C (μM/mM)	37.8 ± 8.2	35.7 ± 7.7		43.2 ± 7.7		34.1 ± 5.1		<0.001	<0.001
ApoA-II/HDL-C (μM/mM) (μmol/mmol)	15.9 ± 4.6	14.8 ± 4.4		19.0 ± 4.1		13.4 ± 2.7		<0.001	<0.001
Cholesterol (mM)	5.65 ± 1.13	5.46 ± 1.08		5.88 ± 1.18		5.75 ± 1.11		<0.001	<0.001
NonHDL-C (mM)	4.33 ± 1.21	4.05 ± 1.14		4.87 ± 1.19		4.13 ± 1.13		<0.001	<0.001
LDL-C (mM)	3.69 ± 1.05	3.51 ± 0.99		4.04 ± 1.04		3.58 ± 1.05		<0.001	<0.001
Triglycerides (mM)	1.16 (0.85–1.68)	1.00 (0.74–1.40)		1.59 (1.14–2.27)		1.09 (0.83–1.42)		<0.001	<0.001
ApoB (g/L)	1.04 ± 0.30	0.96 ± 0.28		1.17 ± 0.32		1.01 ± 0.27		<0.001	<0.001
Glucose (mM)	4.89 ± 1.19	4.67 ± 0.79		5.24 ± 1.58		4.76 ± 0.96		<0.001	<0.001
Creatinine (μM)	83.9 ± 19.5	83.1 ± 14.3		87.8 ± 26.3		80.7 ± 14.5		<0.001	<0.001
UAE (mg/24 h)	9.5 (6.3–17.8)	8.4 (6.0–13.6)		11.8 (7.2–27.1)		9.4 (6.1–17.4)		<0.001	<0.001
eGFR (mL/min/1.73 m^2^)	84.0 ± 15.6	86.5 ± 14.8		81.3 ± 16.1		81.5 ± 15.5		<0.001	<0.001
ApoE (μM)	1.15 ± 0.47	1.09 ± 0.42		1.26 ± 0.57		1.14 ± 0.39		<0.001	<0.001
Paraoxonase/HDL-C (U/mM)	41.7 (32.3–53.8)		39.8 (30.7–50.7)		50.0 (40.5–63.8)		35.0 (28.6–43.5)	<0.001	<0.001
	9		9		9		9		

^a^ For categorical variables, chi-square results showed significant differences (*p* < 0.0001) among the LR, HR1, and HR2 subgroups except for ethanol use (*p* = 0.28); for continuous variables, unadjusted ANOVA revealed statistically significant differences (*p* < 0.0001) among the subpopulations. Subsequent post-hoc testing revealed significant differences (*p* < 0.0001) between populations for all parameters except for cholesterol (HR1 versus HR2, *p* = 0.0018), LDL-C (LR versus HR1, i = 0.035), glucose (LR versus HR1, *p* = 0.017), and apoE (LR versus HR1, *p* = 0.0012) and non-significant results for nonHDL-C (LR versus HR2, *p* = 0.078), triglycerides (LR versus HR2, *p* = 0.90), and UAE (LR versus HR2, *p* = 0.55). ^b^ ANOVA adjusted for age and gender also revealed statistically significant differences (*p* < 0.0001) among the subpopulations. BMI, body mass index; HDL-C, high-density lipoprotein cholesterol; CRP, C-reactive protein; PON1, Paroxonase-1; ApoA-I, apolipoprotein A-I; ApoA-II, apolipoprotein A-II; NonHDL-C, non-HDL lipoprotein cholesterol; LDL-C, low-density lipoprotein cholesterol; ApoB, apolipoprotein B; ApoE, apolipoprotein E; UAE, urinary albumin excretion; eGFR, estimated glomerular filtration rate.

**Table 2:** Spearman correlation coefficients of PON1 with biomarker levels for total, LR, HR1, and HR2 populations.

Parameter	Total Population	Low Risk	High-Risk 1	High-Risk 2
HDL-C	0.20 ***	0.19 ***	0.11 ***	0.15 ***
CRP	−0.05 ***	−0.02	−0.06 **	0.02
ApoA-I	0.19 ***	0.17 ***	0.10 ***	0.15 ***
ApoA-II	0.28 ***	0.26 ***	0.23 ***	0.26 ***
ApoA-I/HDL-C	−0.12 ***	−0.11 ***	−0.04	0.01
ApoA-II/HDL-C (μmol/mmol)	−0.04 ***	−0.03	0.08 ***	0.13 ***
Cholesterol	0.08 ***	0.13 ***	0.09 ***	0.01
NonHDL-C	0.01	0.05 **	0.07 **	−0.03
LDL-C	0.00	0.04 *	0.04	−0.06 *
Triglycerides	0.03 *	0.03 *	0.09 ***	0.13 ***
ApoB	0.00	0.03	0.05 *	−0.02
Glucose	−0.09 ***	−0.09 ***	−0.05 *	−0.07 **
Creatinine	−0.03 **	0.01	−0.04	−0.02
UAE	−0.02	0.02	−0.01	0.01
eGFR	0.02 *	−0.01	0.05 *	0.03
ApoE	0.00	0.04 *	0.04	−0.07 **

* *p* < 0.05; ** *p* < 0.01; *** *p* < 0.001.

**Table 3:** Cox multivariable proportional hazards modeling results for PON1 level giving hazard ratio (HR), 95% confidence interval (95% CI), and *p*-value. The first column lists the population examined (LR, HR1, or HR2) along with parameters adjusted in models. Hazard ratios are per SD unit. For regression calculations, PON1 levels were transformed to a distribution with mean of zero and SD of one.

Population	Model Adjustments	HR	95% CI (86.1% of Subjects)	*p*-Value
LR	unadjusted	0.86	0.75–0.98	0.024
LR	gender, age	0.95	0.82–1.09	0.46
HR1	unadjusted	0.93	0.83–1.04	0.22
HR1	gender, age	1.02	0.91–1.14	0.72
HR2	unadjusted	0.62	0.000001	0.000001
HR2	gender, age	0.72	0.59–0.87	0.0007
HR2	gender, age, UAE	0.70	0.58–0.86	0.0004
HR2	gender, age, UAE, apoB	0.70	0.57–0.85	0.0003
HR2	gender, age, UAE, past CVD	0.71	0.58–0.86	0.0005
HR2	gender, age, UAE, DM	0.71	0.58–0.86	0.0006
HR2	gender, age, UAE, statins	0.69	0.57–0.83	0.0002
HR2	gender, age, UAE, anti-hypertensives	0.68	0.56–0.83	0.0001
HR2	gender, age, UAE, SBP	0.70	0.58–0.85	0.0004
HR2	gender, age, UAE, DBP	0.70	0.58–0.85	0.0004
HR2	gender, age, UAE, smoking	0.70	0.58–0.85	0.0004
HR2	gender, age, UAE, ethanol use	0.69	0.57–0.84	0.0003
HR2	gender, age, UAE, eGFR	0.72	0.59–0.87	0.0009
HR2	gender, age, UAE, past CVD, DM,	0.68	0.55–0.83	0.0003
	apoB, statins, anti-hypertensives			
	smoking, ethanol use, eGFR, SBP, DBP			

**Table 4:** Interaction of PON1 with HDL-related parameters for the HR2 population as assessed by Cox proportional hazards models of cardiovascular event occurrence adjusted for gender, age, UAE, past CVD history, diabetes, apoB, statin use, anti-hypertensive use, smoking, ethanol use, eGFR, SBP, and DBP. Hazard ratios are per SD unit. For regression calculations, continuous variables were transformed to distributions with means of zero and SDs of one.

	Models without Interaction			Models with Interaction		
Parameters	HR	95% CI	*p*	HR	95% CI	*p*
PON1	0.67	0.54−0.83	0.0003	0.76	0.57−1.01	0.059
HDL-C	1.04	0.75−1.45	0.82	1.01	0.72−1.40	0.98
interaction				0.81	0.057−1.16	0.25
PON1	0.69	0.56−0.86	0.0007	0.69	0.54−0.89	0.0036
ApoA-I	0.76	0.58−1.00	0.051	0.76	0.58−1.00	0.054
interaction				0.99	0.77−1.29	0.96
PON1	0.70	0.57−0.88	0.002	0.70	0.57−0.87	0.001
ApoA-II	0.78	0.60−1.02	0.070	0.79	0.61−1.02	0.070
interaction				1.09	0.90−1.31	0.39
PON1	0.66	0.54−0.82	0.0001	0.71	0.55−0.92	0.008
ApoA-I/HDL-C	0.56	0.37−0.85	0.0057	0.59	0.39−0.89	0.013
interaction				1.17	0.87−1.57	0.30
PON1	0.68	0.55–0.84	0.0004	0.81	0.60−1.08	0.15
ApoA-II/HDL-C	0.63	0.40−1.00	0.051	0.65	0.41−1.03	0.065
interaction				1.30	0.94−1.80	0.12
PON1	0.66	0.53−0.82	0.0002	0.64	0.51−0.80	0.0001
ApoE	0.82	0.60−1.12	0.21	0.85	0.62−1.16	0.30
interaction				1.77	1.29−2.41	0.0003
